# Early transcriptome changes induced by the Geminivirus C4 oncoprotein: setting the stage for oncogenesis

**DOI:** 10.1186/s12864-021-07455-y

**Published:** 2021-03-02

**Authors:** Carl Michael Deom, Magdy S. Alabady, Li Yang

**Affiliations:** 1 Department of Plant Pathology, University of Georgia, Athens, GA USA; 2 Department of Plant Biology, University of Georgia, Athens, GA USA

**Keywords:** C4 protein, Curtovirus, RNA-seq, Hormone homeostasis, Cell wall homeostasis, BR signaling pathway-dependent, BR signaling pathway-independent

## Abstract

**Background:**

The Beet curly top virus C4 oncoprotein is a pathogenic determinant capable of inducing extensive developmental abnormalities. No studies to date have investigated how the transcriptional profiles differ between plants expressing or not expressing the C4 oncoprotein.

**Results:**

We investigated early transcriptional changes in *Arabidopsis* associated with expression of the Beet curly top virus C4 protein that represent initial events in pathogenesis via a comparative transcriptional analysis of mRNAs and small RNAs. We identified 48 and 94 differentially expressed genes at 6- and 12-h post-induction versus control plants. These early time points were selected to focus on direct regulatory effects of C4 expression. Since previous evidence suggested that the C4 protein regulated the brassinosteroid (BR)-signaling pathway, differentially expressed genes could be divided into two groups: those responsive to alterations in the BR-signaling pathway and those uniquely responsive to C4. Early transcriptional changes that disrupted hormone homeostasis, 18 and 19 differentially expressed genes at both 6- and 12-hpi, respectively, were responsive to C4-induced regulation of the BR-signaling pathway. Other C4-induced differentially expressed genes appeared independent of the BR-signaling pathway at 12-hpi, including changes that could alter cell development (4 genes), cell wall homeostasis (5 genes), redox homeostasis (11 genes) and lipid transport (4 genes). Minimal effects were observed on expression of small RNAs.

**Conclusion:**

This work identifies initial events in genetic regulation induced by a geminivirus C4 oncoprotein. We provide evidence suggesting the C4 protein regulates multiple regulatory pathways and provides valuable insights into the role of the C4 protein in regulating initial events in pathogenesis.

**Supplementary Information:**

The online version contains supplementary material available at 10.1186/s12864-021-07455-y.

## Background

Plant virus proteins are adept at co-opting cellular machinery and metabolic pathways to alter the host physiology to benefit the virus life cycle [1]. One such virus protein is the small C4 protein (~ 10 kDa) (*AC4* in geminiviruses with bipartite genomes) encoded by some members of the *Geminiviridae* [2]. Viruses within the *Geminiviridae* family cause a variety of economically important diseases in crop plants worldwide [3]. Studies on the role of the C4/AC4 proteins from members of the *Curtovirus* and *Begomovirus* genera suggest that the proteins have a diverse set of functions by which they modulate pathogenesis.

Some geminivirus C4/AC4 proteins have been shown to induce oncogenesis during virus infection [4] and when expressed ectopically [4–8]. The oncogenic nature of C4/AC4 proteins has been shown to, at least in part, result from their ability to interfere with the function of shaggy-like protein kinases [8–10]. In *Arabidopsis*, seven shaggy-like protein kinases (AtSKs) of the ten-member multigene family have been implicated in negatively regulating the brassinosteroid (BR) signaling pathway (BRSP) [11–14]. BR is a steroid hormone that functions as a master regulator of plant development, growth and adaption to stress [15]. AtSKs have also been implicated in crosstalk between the BRSP and other hormone signaling pathways, biotic and abiotic stress responses, root and stomata development, flower development, xylem differentiation, phloem development, and pattern-triggered immunity [13, 14, 16–19].

AtSKs regulate the closely related BRI1-EMS SUPPRESSOR 1 (BES1) and BRASSINAZOLE-RESISTANT 1 (BZR1) transcription factors, which are pivotal in the BRSP [14]. In the absence of BR, AtSKs hyperphosphorylate and inactivate BES1 and BZR1. In the presence of BR, the hormone binds to the cell surface receptor kinase BRASSINOSTEROID INSENSITIVE 1 (BRI1) and co-receptor BRASSINOSTEROID INSENSITIVE 1-ASSOCIATED RECEPTOR KINASE 1 (BAK1), initiating a signaling cascade that results in the negative regulation of AtSKs and the activation of BES1/BZR1 transcription factors. BZR1/BES1 regulate a large number of BR-responsive genes and repress BR biosynthetic genes [15, 20–25]. Activated BES1/BZR1 transcription factors coordinate a complex multisignal regulatory network controlling growth and development [26].

The C4 protein of the curtovirus Beet curly top virus (BCTV) binds to the 7 AtSKs implicated in BR signaling. The protein interferes with the function of the AtSKs and sequesters the kinases to the plasma membrane (PM; 9). Chemical inhibition of the same 7 AtSKs with bikinin induces hyperplasia that phenocopies symptoms induced by C4, suggesting hyperplasia may, at least in part, be due to C4 modulating the function of some combination of the 7 AtSKs [9]. Consistent with this, the C4 protein of the begomovirus Tomato leaf curl Yunnan virus (TLCYnV) also was shown to interact with *N. benthamiana* shaggy-like protein kinase η (NbSKη) and sequester the kinase to the PM [8]. This interaction was suggested to impair NbSKη directed degradation of NbCycD1;1, resulting in abnormal cell division [8]. Interactions between three additional begomovirus C4 proteins and shaggy-like protein kinases have also been confirmed although their role in pathogenesis is not known [10, 27, 28].

A number of other C4/AC4-host protein interactions have been identified. A curtovirus C4 protein was shown to bind to CLAVATA 1 (CLV1) [29]. Two begomovirus C4/AC4 proteins were shown to interact with CLV1-type PM receptor-like kinases BARELY ANY MERISTEM 1 and 2 (BAM1, BAM2) [30, 31], interfering with their ability to regulate cell-to-cell movement of RNAi [30]. CLV1, BAM1 and BAM2 are required for shoot apical meristem homeostasis, as well as vascular tissue, anther and root development [32]. In addition, begomovirus C4 proteins have been shown to interact with S-ADENOSYL METHIONINE SYNTHETASE and AGONAUTE 4, proteins that modulate gene silencing [33, 34], and HYPERSENSITIVE INDUCED REACTION 1 (HIR1), impairing the HIR1-mediated hypersensitive response [35].

Transcriptional analyses of geminivirus-infected plants have been shown to impact defense/immune responses, hormone homeostasis, the cell cycle and autophagy [36–39]. Ectopically expressed BCTV C4 leads to a severe developmental phenotype characterized by the loss of meristem function, prolific cell division and loss of cell-type differentiation [6]. These ectopically expressed changes are similar to those observed in the vascular tissue of infected plants, suggesting that ectopically expressed C4 recapitulates C4 pathogenesis observed during infection. To develop a better understanding of the role of the C4 protein in pathogenesis in the absence of BCTV infection, we performed a comparative transcriptional analysis from transgenic *Arabidopsis* plants expressing the BCTV C4 protein under the regulatory control of an inducible promoter relative to non-induced plants. To identify changes in C4-induced gene expression that are expected to represent initial events in pathogenesis, we chose early times post-C4 induction (6 and 12 h) as opposed to later times where more complex gene expression patterns likely would be composed of both direct and indirect changes to the transcriptome.

We observed that C4-induced transcriptional changes disrupted hormone homeostasis that were indicative of the protein regulating the BRSP. Other transcriptome changes suggest that C4 interferes with cell development, cell wall homeostasis, redox homeostasis and lipid transport in a C4-induced BRSP-independent manner. The results provide insights into the multifunction role of the BCTV C4 protein in virus infection and pathogenesis.

## Results

### Transcriptional analysis design

**Fig. 1:**
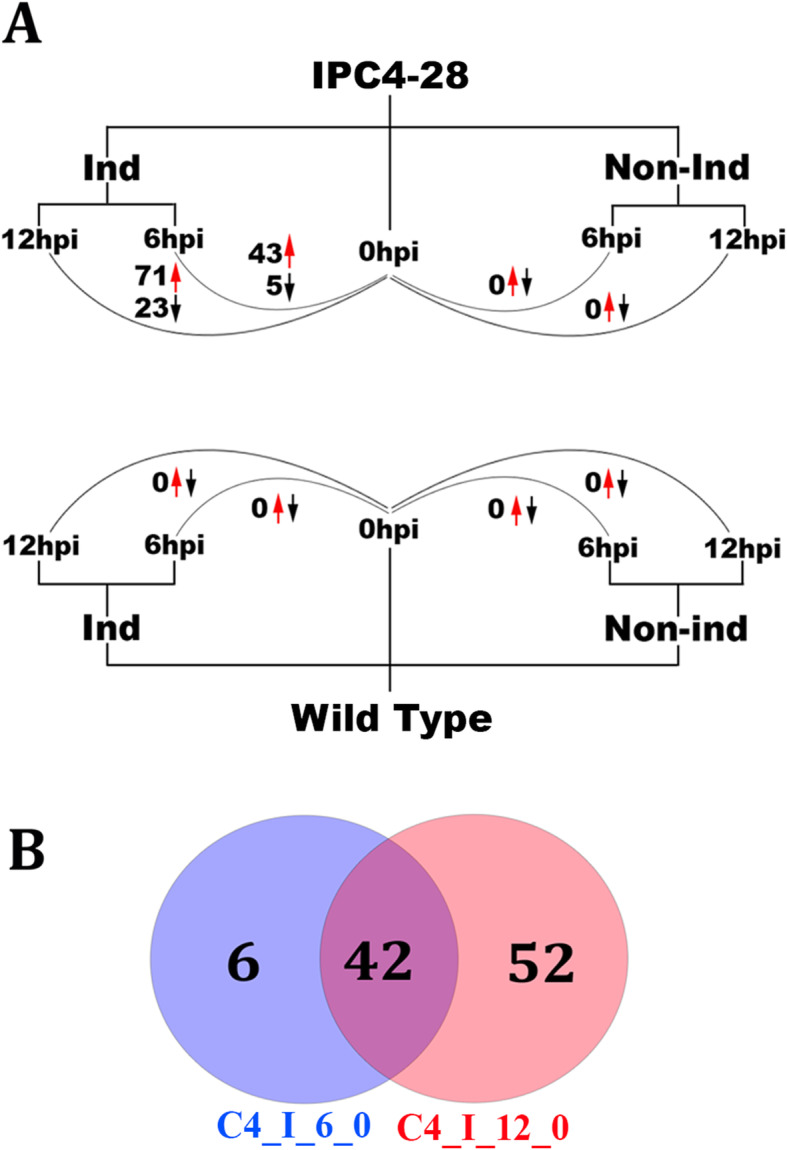
**a** Schematic of the experimental design and comparison of the number of differentially expressed up- (up arrows) and down-regulated (down arrows) genes at 6- and 12-h post-induction (hpi) of the BCTV *C4* gene. Transgenic line IPC4–28 expresses the Beet curly top virus *C4* gene under regulatory control of a ß-estradiol (ß-est) inducible promoter. Wild Type, Sei-O. Ind, Induced. Nonind, Noninduced. **b** Venn diagram showing the number of differentially expressed genes under different experimental conditions. C4_I_6_0 (blue), number of C4-induced differentially expressed genes at 6-hpi versus 0-hpi. C4_I_12_0 (orange), number of C4-induced differentially expressed genes at 12-hpi versus 0-hpi. Overlap (purple), number of C4-induced differentially expressed genes at 6-and 12-hpi versus 0-hpi

To begin understanding gene expression changes induced by the BCTV C4 protein, we performed RNA-seq analysis on RNA extracted from seedlings of *Arabidopsis* line IPC4–28 expressing or not expressing the C4 protein at 6- and 12-h post-induction (hpi) or post-mock induction (Fig. 1a). IPC4–28 is a transgenic line that expresses the BCTV C4 protein under the regulatory control of a ß-estradiol (ß-est) inducible promoter. Induction with ß-est results in near synchronized expression of the C4 protein, which is detectable by Western blot analysis as early as 6-hpi [6]. Initial symptoms of an abnormal development phenotype occurred in IPC4–28 seedlings geminated in liquid media in the presence of 10 μM ß-est as early as 2-days post-induction [6]. The noninduced IPC4–28 seedlings were unaltered and phenotypically identical to the wild type (WT) seedlings under light microscopy looking at cotyledon development and light microscopy and scanning electron microscopy looking at shoot and root apical meristem development [6]. We hypothesized that early effects on host gene expression caused by C4-host protein interactions would give insights into initial events leading to C4 induced pathogenesis. This would minimize complexities resulting from secondary down-stream gene expression changes that result with time following C4 expression or that develop from expression of other viral proteins during virus infection. RNA-seq resulted in a total of 576.0 million reads that passed quality control for 30 libraries, with an average of 19.2 million reads per library (Supplementary Fig. S1, Additional file 1). Of these reads, 85–88% (three library replicates/treatment) mapped to the *Arabidopsis* reference genome.

### Differentially expressed genes following C4 induction

**Fig. 2:**
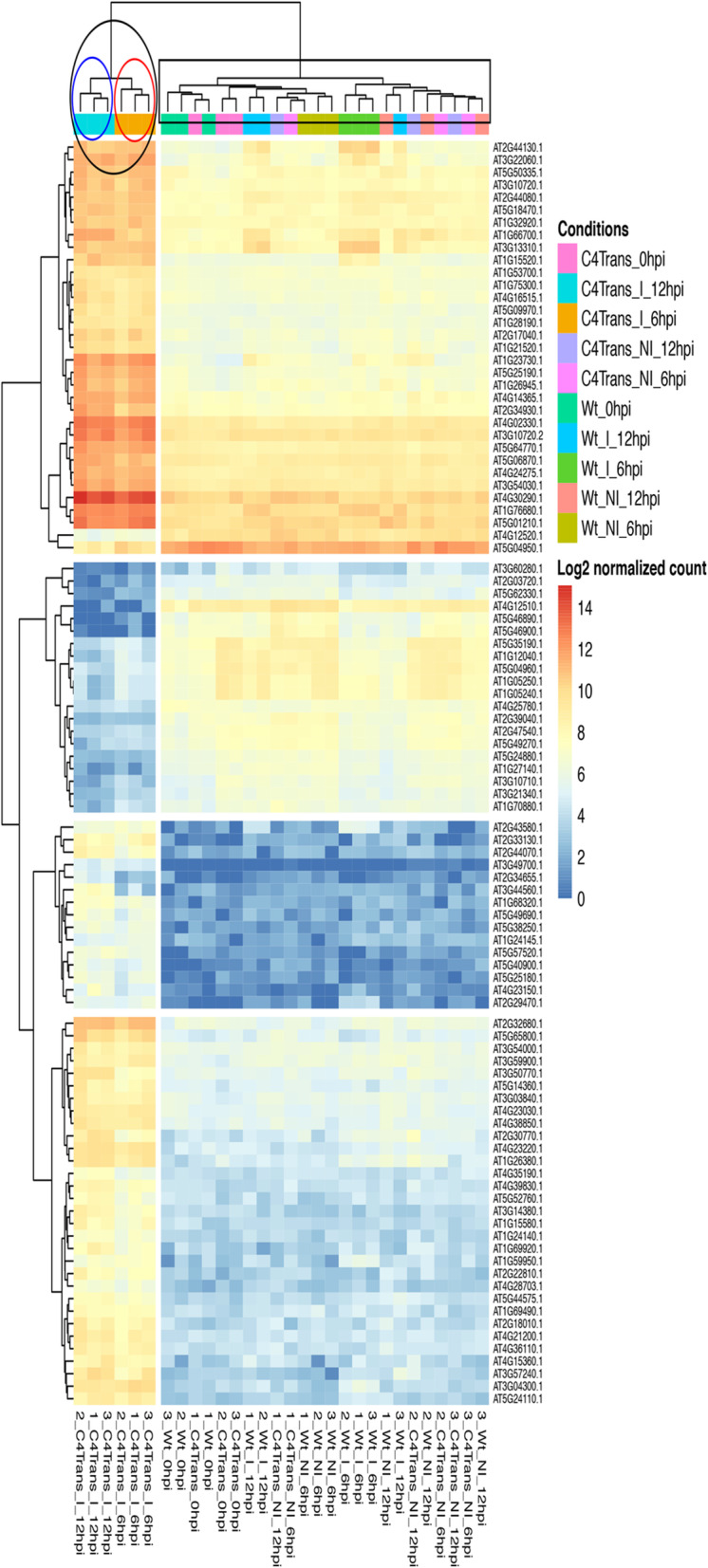
Gene heat map showing hierarchical clustering of differentially expressed genes based on color-coded expression levels. Conditions indicate seedlings that were induced or noninduced at 6- and 12-hpi. C4 Trans, IPC4–28 seedlings. WT, Sei-0 seedlings. I, induced. NI, noninduced. Cluster in black oval represent IPC4–28 seedlings induced at 6- and 12-hpi. Subgroup in red  oval represents IPC4–28 seedlings induced at 6-hpi. Subgroup in blue  oval represents IPC4–28 seedlings induced at 12-hpi. The expression values are represented in the Log2 scale of normalized counts of gene expression

At 6-hpi, 48 DE genes were detected in induced IPC4–28 seedlings with 43 up-regulated (90%) and 5 down-regulated (10%). At 12-hpi, 94 DE genes were detected in induced IPC4–28 seedlings with 71 up-regulated (76%) and 23 down-regulated (24%) (Fig. 1b, Additional file 2). Six DE genes were distinct in seedlings at 6-hpi, 52 were distinct at 12-hpi and 42 were common to both time points (Fig. 1b). Of the DE genes in common at 6-hpi and 12-hpi, 37 (88%) were up regulated and expressed at 6-hpi > 78% of the levels detected at 12-hpi. Five were down regulated and repressed at 6-hpi > 84% of the levels detected at 12-hpi. Therefore, regulation of transcriptional differences detected at 6-hpi were generally maintained and amplified at 12-hpi. In control experiments with Sei-0 wild-type seedlings, no ß-est induced DE genes were detected in WT seedlings when compared to noninduced WT seedlings at 6- and 12-hpi (Fig. 1a), indicating that ß-est did not induce any detectable DE genes under the parameters used. Similarly, the only DE genes detected when comparing induce IPC4–28 seedlings to induced WT seedlings in the presence of 10 μM ß-est were the same DE genes detected when comparing induced IPC4–28 seedlings to noninduced IPC4–28 seedlings. To better understand the transcriptional dynamics from 0- through 12-hpi, a heatmap showing changes in the expression patterns of the DE genes is shown in Fig. 2. DE genes fell into 2 major clusters based on their patterns of expression and were distinct for induced IPC4–28 seedlings at 6- and 12-hpi relative to mock-induced IPC4–28 seedlings or induced and mock-induced wild-type seedlings. The group represented by induced IPC4–28 seedlings was composed of 2 subgroups separately representing induced IPC4–28 seedings at 6-hpi and at 12-hpi. This finding was supported by the principle component analysis (PCA) illustrated in Supplementary Fig. S2 (Additional file 1), which shows that replicates from 6- and 12-hpi transgenic induced samples are clustered at a distance from all other replicates, indicating a large variance in the expression of these two conditions comparing to the others.

The number of C4 mRNA reads in non-induced IPC4–28 seedlings at 0-, 6-, and 12-hpi were at trace levels compared to levels in induced IPC4–28 seedlings (Additional file 2). For example, there was an average of 11,349 C4 mRNA reads in induced IPC4–28 samples at 12-hpi and an average of 22 C4 mRNA reads in noninduced IPC4–28 samples at 12 hpmi. No DE genes were detected under our parameters that would have resulted from trace amounts of C4 mRNA due to promoter leakage when comparing noninduced IPC4–28 and noninduced WT samples. However, we cannot absolutely exclude the possibility that some trace amount of C4 is present in noninduced IPC4–28 plants that might have a small effect on the transcriptome and was nondetectable under the parameters used.

### Validation of differentially expressed genes detected by RNA-seq

**Fig. 3:**
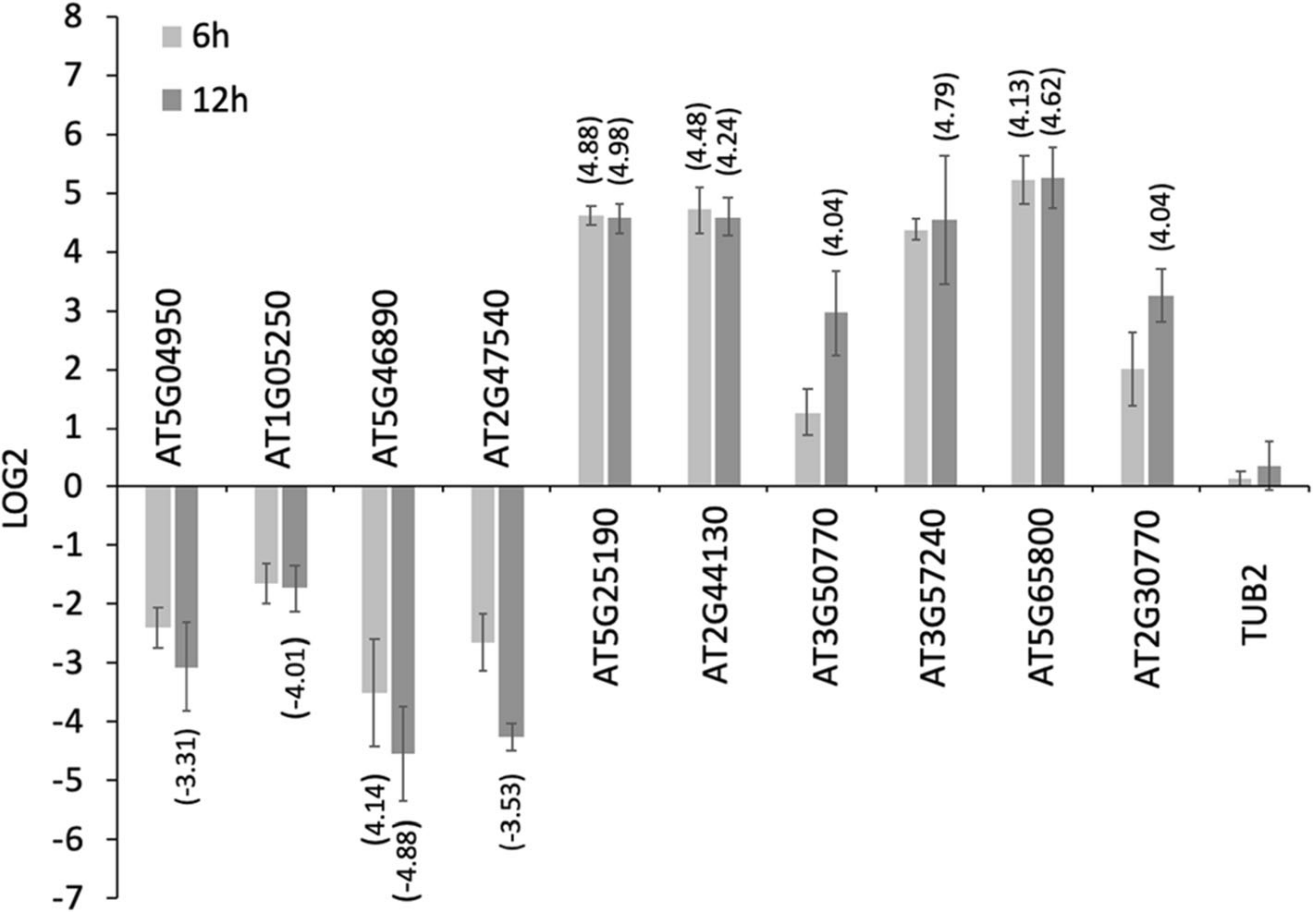
Validation of RNA-seq expression results by comparison with quantitative reverse transcription-PCR (RT-qPCR) data from the same RNA samples. Comparison of Log2 fold changes of 10 genes at 12-hpi. *TUB2* was used as a control that was not differentially expressed in the RNA-seq experiments. *MON1* was used as an endogenous housekeeping control. C4-induced transcriptional changes were normalized to gene expression in noninduced seedlings. Columns represent Log2 fold changes from RT-qPCR between ß-est treated and mock treated samples. Numbers in parenthesis indicate induced Log2 fold changes from RNA-seq expression data (Additional file 2) for direct comparison with RT-qPCR data. Log2 fold changes of 4 of the 10 genes that were differentially expressed at 6-hpi are also shown. Error bars indicate standard deviations from three biological replicates

To verify the RNA-seq data, 10 DE genes (4 down and 6 up-regulated) were validated by reverse transcription-quantitative PCR (RT-qPCR). The 4 down-regulated genes were: *AT5G04950* (*NICOTIANAMINE SYNTHASE 1, NAS1*), *AT1G05250* (*PEROXIDASE 2, PRX2*), *AT5G46890* (lipid transfer protein) and *AT2G47540* (*POLLEN OLE E 1 ALLERGEN* extensin family protein). The 6 up-regulated genes were: *AT3G50770* (*CALMODULIN-LIKE 41 PROTEIN*, *CML41*), *AT3G57240 (Β-1,3-GLUCANASE 3*), *AT5G65800* (*1-AMINOCYCLOPROPANE-1-CARBOXYLATE SYNTHASE 5*, *ACS5*), *AT2G30770* (*CYTOCHROME P71A*13), *AT5G25190* (*ETHYLENE-RESPONSE TRANSCRIPTION FACTOR 003*), *AT2G44130* (*KELCH-DOMAIN CONTAINING F-BOX PROTEIN 39*). *AT5G62690* (*TUBULIN βCHAIN 2, TUB2*) was also analyzed as a control gene not regulated by ß-est. Fold changes were compared for the 11 genes from RNA-Seq and RT-qPCR (Fig. 3). A Pearson correlation coefficient of *R*^*2*^ = 0.95, *P* < 0.0001) indicates a high correlation between the two methods and validates the RNA-seq data.

### C4 regulated DE genes that were BR-signaling pathway dependent and BR-signaling pathway independent

To determine the extent to which C4 regulates genes in the BRSP by inhibiting AtSKs, we compared the C4-responsive DE genes identified at 12-hpi with databases of BR-responsive genes and DE genes in *bes1-D* and *bzr1-1D* gain-of-function mutants (24, 25, Table 1 and Additional file 3). *Bes1-D* and *bzr1-1D* are constitutively active mutants [21, 22]. At 12-hpi, 43 (61%) of the C4 up-regulated DE genes were previously shown to be responsive to BR and/or one or both of the gain-of-function mutants, while 28 (39%) of the up-regulated DE genes were responsive to C4, but not to BR, *bes1-D* and/or *bzr1-1D*. Of the 23 down-regulated DE genes responsive to C4 at 12-hpi, 20 (87%) were regulated independent of the BRSP. Of these 20 C4 down-regulated DE genes, 4 have previously been shown to be up-regulated by BR and/or the *bzr1-1D* gain-of-function mutant and 16 were only responsive to C4. The results indicate that expression of C4 has a direct effect on regulating the BRSP (BRSP-dependent response). However, a subset of C4-regulated DE genes are uniquely responsive to C4 and independent of the BRSP (BRSP-independent).

**Table 1:** Number of C4-regulated genes induced in a brassinosteroid-signaling pathway dependent or independent manner at 12-hpi

DE genes	Dependent	Independent
Up-regulated	43 (61%)	28 (39%)
Down-regulated	3 (13%)	20 (87%)

### Gene ontology analysis

**Fig. 4:**
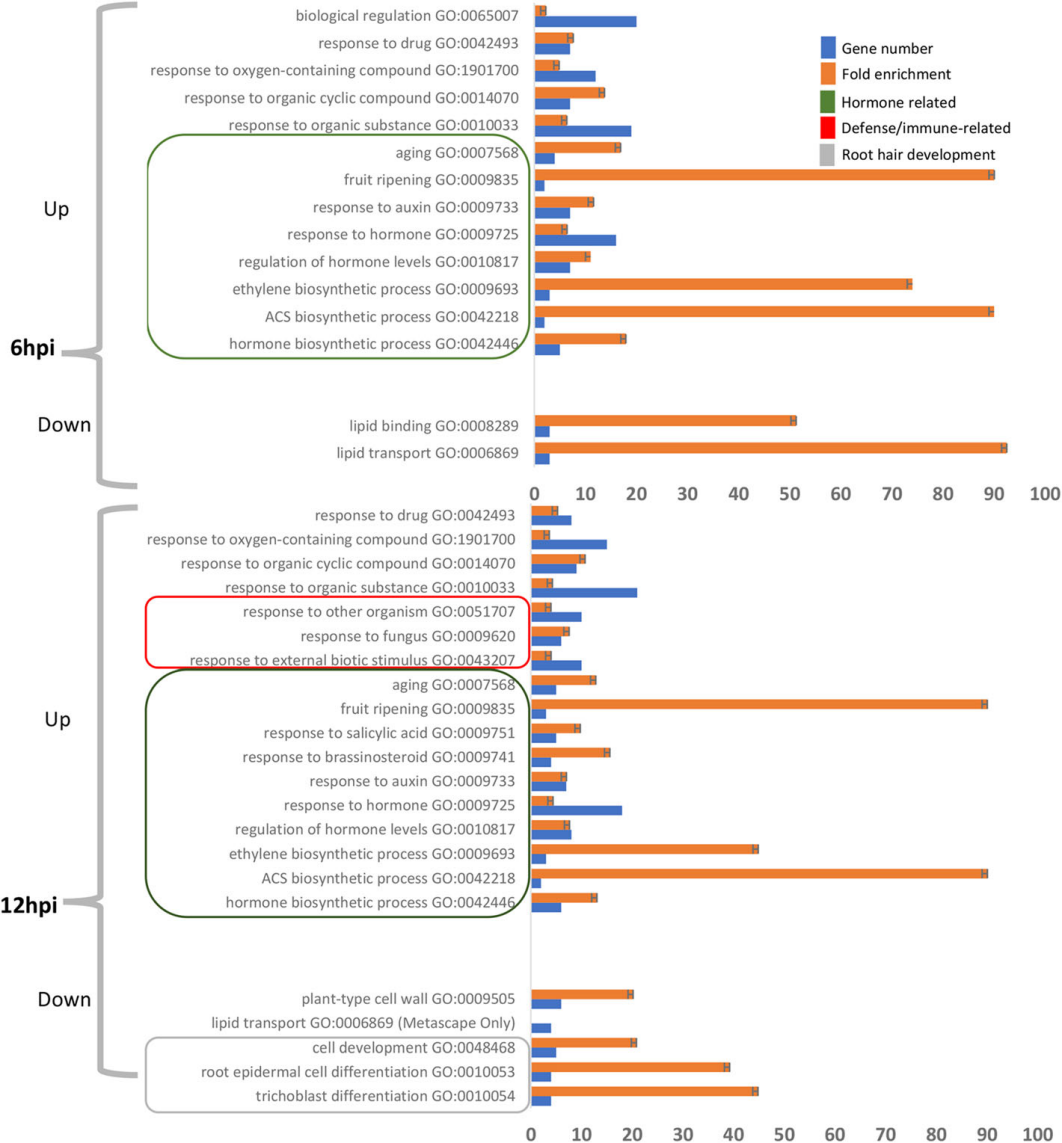
GO categories representing over or under-represented genes differentially expressed in C4 expressing seedlings relative to non-expressing seedlings. Bars represent the percentage of regulated genes in addition to those expected by chance in the Arabidopsis reference (Enrichment test). GO categories were organized into those representing up-regulated genes or those representing down-regulated genes. Blue bars indicate gene numbers and orange bars indicated fold enrichment. Categories enclosed in green rectangles are hormone related, those enclosed in the red rectangle are defense/immune related and those enclosed in the grey rectangle are cell development related. All categories had false discovery rates with *P* < 0.05

To gain insights into biological processes the C4 protein modulates at early time points following induction, gene ontology (GO) enrichment analysis was utilized to identify GO terms over- or under-represented for DE genes identified in induced IPC4–28 seedlings at 6- and 12-hpi (Fig. 4). At 6-hpi, GO enrichment identified 13 categories from up-regulated DE genes involved in biological processes, including 8 associated with hormone-related processes. GO enrichment identified 2 related categories from down-regulated DE genes associated with lipid transport and lipid binding. Of the DE genes identified at 6-hpi, 65% were captured by GO enrichment categories. At 12-hpi, GO enrichment identified 17 categories from up-regulated DE genes, including 10 associated with hormone-related processes and 3 associated with pathogen defense. Five GO categories were identified from down-regulated DE genes, including 3 associated with cell development and 1 each associated with lipid transfer and cell wall processes (Fig. 4). At 12-hpi, 52% of the DE genes are members in GO enrichment categories.

### Early events in C4 expression associated with hormone homeostasis

C4 up-regulated DE genes associated with hormone homeostasis, included genes involved in hormone biosynthesis and the regulation of hormone levels as well as DE genes responsive to auxin, BR and salicylic acid (Additional file 4 and Table 2). At 6- and 12-hpi, 40% (19 of 48) and 24% (23 of 94), respectively, of all DE genes were associated with hormone homeostasis. This indicates that much of the initial response to C4 resulted in up-regulated expression of genes that have an effect on hormone homeostasis or respond to alterations in hormone homeostasis, a trend seen at 6-hpi and increased at 12-hpi. All DE genes affecting hormone homeostasis at 12-hpi were responsive to BR and/or one or both of the *bes1-D* and *bzr1-1D* gain-of-function mutants with the exception of *1-AMINOCYCLOPROPANE-1-CARBOXYLATE SYNTHASE 9* (*ACS9*), *LONELY GUY 5* (*LOG5)* and *PARAXANTHINE METHYLTRANSFERASE 1* (*PXMT1*) (Table 2 and Additional file 3). The expression of the *ABC TRANSPORTER G FAMILY 40 protein* (*ABCG40*) was differentially responsive to C4 in that the gene is down-regulated in *bes1-D* but up-regulated following C4 expression. These results suggest that most of the hormone-related responses can be contributed to the role of the C4 protein in regulating the BRSP.

**Table 2:** Gene ontology (GO) terms indicating over-represented, up-regulated genes in hormone homeostasis at 12-h post-induction

GO terms	*P*-value^*^	FDR^**^	Fold enrichment	Gene^a^ symbol	Gene name (Gene ID)
Hormone biosynthesis (GO:0042446)	8.25E-06	6.10E-03	13.02	*ACS4*	*1-aminocyclopropane-1-carboxylate synthase 4 (AT2G22810)*
				*ACS5*	*1-aminocyclopropane-1-carboxylate synthase 5 (AT5G65800)*
				***ACS9***	*1-aminocyclopropane-1-carboxylate synthase 9 (AT3G49700)*
				*GA2OX8*	*Gibberellin 2-oxidase (AT4G21200)*
				***LOG5***	*Lysine decarboxylase protein 5 (AT4G35190)*
				*MYB62*	*R2R3-MYB transcription family 62 (AT1G68320)*
Regulation of hormone levels (GO:0010817)	1.12E-05	7.37E-03	7.64	*ACS4*	*1-aminocyclopropane-1-carboxylate synthase 4 (AT2G22810)*
				*ACS5*	*1-aminocyclopropane-1-carboxylate synthase 5 (AT5G65800)*
				***ACS9***	*1-aminocyclopropane-1-carboxylate synthase 9 (AT3G49700)*
				*GA2OX8*	*Gibberellin 2-oxidase (AT4G21200)*
				***LOG5***	*Lysine decarboxylase protein 5 (AT4G35190)*
				*MYB62*	*R2R3-MYB transcription family 62 (AT1G68320)*
				*RGF6*	*Root meristem growth factor 6 (AT4G16515)*
				*RGF9*	*Root meristem growth factor 9 (AT5G64770)*
Response to auxin (GO:0009733)	6.97E-05	2.06E-02	7.01	*ACS4*	*1-aminocyclopropane-1-carboxylate synthase 4 (AT2G22810)*
				*IAA5*	*Indole − 3-acetic acid inducible 5 (AT1G15580)*
				*SAUR9*	*Small auxin upregulated RNA 9 (AT4G36110)*
				*SAUR10*	*Small auxin upregulated RNA 10 (AT2G18010)*
				*SAUR15*	*Small auxin upregulated RNA 15 (AT4G38850)*
				*SAUR27*	*Small auxin upregulated RNA 27 (AT3G03840)*
				*XTH19*	*Xyloglucan endotransglucosylase/hydrolase protein 19 (AT4G30290)*
Response to brassinosteroid (GO:0009741)	1.51E-04	3.89E-02	15.54	*ARL*	*ARGOS-like protein (AT2G44080)*
				*BSK6*	*Brassinosteroid-signaling kinase 6 (AT3G54030)*
				*PME41*	*Pectin methylesterase 41 (AT4G02330)*
				*SAUR10*	*Small auxin upregulated RNA 10 (AT2G18010)*
Response to salicylic acid (GO:0009751)	1.87E-04	4.25E-02	9.71	***ABCG40***	*ABC transporter G family member 40 (AT1G15520)*
				*MYB62*	*R2R3-MYB transcription family 62 (AT1G68320)*
				***PXMT1***	*Paraxanthine methyltransferase 1 (AT1G66700)*
				*OPR1*	*12-oxophytodienoate reductase 1 (AT1G76680)*
				*WRKY30*	*WRKY transcription factor 30 (AT5G24110)*
Response to hormone (GO:0009725)	9.26E-08	1.37E-04	4.37	*ACS4*	*1-aminocyclopropane-1-carboxylate synthase 4 (AT2G22810)*
				*ACS5*	*1-aminocyclopropane-1-carboxylate synthase 5 (AT5G65800)*
				***ABCG40***	*ABC transporter G family member 40 (AT1G15520)*
				*ARL*	*ARGOS-like protein (AT2G44080)*
				*BSK6*	*Brassinosteroid-signaling kinase 6 (AT3G54030)*
				*CRRSP38*	*Cysteine-rich repeat secretory protein 38 (AT3G22060)*
				*ERF003*	*Ethylene-responsive transcription factor 003 (AT5G25190)*
				*IAA5*	*Indole − 3-acetic acid inducible 5 (AT1G15580)*
				*MYB62*	*R2R3-MYB transcription family 62 (AT1G68320)*
				*OPR1*	*12-oxophytodienoate reductase 1 (AT1G76680)*
				*PME41*	*Pectin methylesterase 41 (AT4G02330)*
				***PXMT1***	*Paraxanthine methyltransferase 1 (AT1G66700)*
				*SAUR9*	*Small auxin upregulated RNA 9 (AT4G36110)*
				*SAUR10*	*Small auxin upregulated RNA 10 (AT2G18010)*
				*SAUR15*	*Small auxin upregulated RNA 15 (AT4G38850)*
				*SAUR27*	*Small auxin upregulated RNA 27 (AT3G03840)*
				*WRKY30*	*WRKY transcription factor 30 (AT5G24110)*
				*XTH19*	*Xyloglucan endotransglucosylase/hydrolase protein 19 (AT4G30290)*

^*^P-values < 0.05

^**^FDR, false discovery rate, with *P* < 0.05

^a^Gene symbols in bold print indicate genes uniquely responsive to C4

### Early events in C4 expression associated with host defense and redox homeostasis

The up-regulation of DE genes under the GO term *response to salicylic acid* observed at 12-hpi may suggest initial events in a salicylic acid-directed pathogen-related response induced by C4 (Table 2). In addition, GO analysis identified up-regulated DE genes associated with host defense (Table 3). Five of the 10 DE genes were significantly up-regulated at 6 hpi. Four additional DE genes that respond to pathogen infection were upregulated, but not captured by the GO enrichment analysis; CML41 (*AT3G50770*, facilitates rapid callose deposition at plasmodesmata when upregulated by bacterial infection [40];, *FAD-LINKED OXIDOREDUCTASE 1* (*AT1G26380,* involved in biosynthesis of a cyanogenic phytoalexin and distinct from the canonical camalexin pathway, required for inducible pathogen defense [41];, *METALLOENDOPROTEINASE 3-MMP* (*AT1G24140,* contributes to pattern-triggered immunity by fungi in *Arabidopsis* [42]; and *POLYGALACTURONASE-INHIBITING PROTEIN 2* (*AT5G06870,* an inhibitor of fungal polygalacturonase [43];. Nine of the 14 DE genes are up-regulated by BR and/or one or both *bes1-D* and *bzr1-1D* gain-of-function mutants (Additional file 3). Three DE genes were uniquely responsive to C4, *INDOLEACETALDOXIME DEHYDRATASE* (*AT2G30770,* cytochrome P450), *AT2G43580* (an endochitinase), and *PXMT 1*. Interestingly, 2 DE genes, *CML41* and *ABCG40*, were differentially responsive to C4 relative to the BRSP, being up-regulated following C4 expression, but down-regulated in *bes1-D* (Additional file 3). The only pathogenesis-related protein responsive to C4 was PEROXIDASE 1 (PRX1, also known as PR9), which was repressed at 12 hpi. Since redox buffering plays a role in plant defenses against pathogens, including viruses [44], we screened annotated DE genes potentially associated with redox buffering. Sixteen DE genes were identified with 11 being responsive to C4, but not to BR, *bes1-D* and/or *bzr1-1D* (Table 4; Additional file 3), suggesting C4 expression might disrupt redox homeostasis in a manner unique to C4 and independent of the BRSP.

**Table 3:** Disease Resistance: Gene ontology enrichment analysis of over-represented, up-regulated genes at 12 h post-induction

GO terms	*P*-value	FDR^*^	Fold enrichment	Gene symbol^a^	Encoded protein name (Gene ID)
Response to other organisms (GO:0051707)	2.25E-04	4.75E-02	3.93	–	Disease resistance family protein/LRR family protein (*AT2G34930*) ^***^
				***CYP71A13***	Indoleacetaldoxime dehydratase (Cytochrome P450) (*AT2G30770*) ^***^
				***–***	Endochitinase (*AT2G43580*) ^***^
				*RLP23*	Receptor-like protein 23 (*AT2G32680*) ^***^
				*PME41*	Pectin methylesterase 41 (*AT4G02330*)^***^
				***PXMT1***	Paraxanthine methyltransferase 1 (*AT1G66700***)** ^***^
				*BG3*	Glucan endo-1-3-beta-glucosidase BG3 (*AT3G57240*)
				*CRK7*	Cysteine-rich receptor-like protein kinase 7 (*AT4G23150*)
				*CRK14*	Cysteine-rich receptor-like protein kinase 14 (*AT4G23220*)
				***ABCG40***	ABC transporter G family 40 (*AT1G15520*)

^*^FDR false discovery rate, value has a *P* < 0.05

^***^Significantly enriched in the GO category *response to fungi* (GO:0009620, *P*-value = 1.63E-04, FDR = 3.86E-02, Fold enrichment = 7.52)

^a^Gene symbols in bold print indicate genes uniquely responsive to C4

**Table 4:** C4-induced genes associated with redox buffering at 12-hpi

Gene	Gene symbol^a^	Encoded protein name	Log 2-fold changes^**^
*AT2G33130*	***RALF18***	Rapid Alkalinization Factor 18	5.80
*AT3G44560*	***FAR8***	Fatty acid reductase 8	5.02
*AT2G29470*	***GSTU3***	Glutathione-S-transferase TAU 3	4.45
*AT5G25180*	***CYP71B14***	Cytochrome P450	4.30
*AT4G39830*	*Cupredoxin superfamily*	Cupredoxin superfamily protein	4.29
*AT4G15360*	*CYP705A3*	Cytochrome P450	4.28
*AT1G69920*	***GSTU12***	Glutathione-S-transferase TAU 12	4.19
*AT1G26380*	*FOX1*	FAD-liked oxidoreductase 1	3.99
*AT5G09970*	*CYP78A7*	Cytochrome P450	3.20
*AT1G76680*	*OPR1*	12-Oxophytodienoate reductase 1	3.15
*AT1G75300*	***isoflavone reductase-like***	isoflavone reductase-like	2.96
*AT3G60280*	***UCC3***	Uclacyanin 3	−3.91
*AT2G39040*	***PER24***	Peroxidase 24	−3.93
*AT1G05240*	***PER1***	Peroxidase 1	−4.01
*AT1G05250*	***PER2***	Peroxidase 2	−4.01
*AT1G27140*	***GSTU14***	Glutathione-S-transferase TAU 14	−4.06

^**^All log2 fold changes had adjusted *p*-values < 0.05

^a^Gene symbols in bold print indicate genes uniquely responsive to C4

### Down-regulated differentially expressed genes uniquely responsive to C4

At 6- and 12-hpi, repressed DE genes were enriched in the GO categories *lipid transport* and *lipid binding* (Table 5). The most significantly down-regulated DE genes at 6- and 12-hpi were the lipid transfer proteins (LTP) *AZELAIC ACID INDUCED 5* (*AZI5*), *AT5G46890* and *AT5G46900*. *AT5G46890* and *AT5G46900* are paralogs of *AZI5*. A fourth LTP, *AT4G12520* (*AZI6*) was also significantly down-regulated at 12-hpi and represents a recent gene duplication of *AZI5*, encoding an identical protein, albeit the promoters are differently regulated. Interestingly, the 4 LTPs are differentially responsive to C4 and BR and *bzr1-1D*. The LTPs are down-regulation by C4, while *AZI5* and *AZI6* are up-regulated by BR and all 4 of the LTPs are up-regulated in the *bzr1-1D* gain-of-function mutant (Additional file 3).

**Table 5:** Gene ontology enrichment analysis of down-regulated genes at 6- and 12-h post induction

GO terms	*P*-value^*^	FDR^**^	Fold enrichment	Gene symbol^a^	Encoded protein name (Gene ID)
Down-regulated genes at 6 and 12 h post-induction					
Lipid transport (GO:0006869, biological process)	2.80E-06	1.65E-02	92.45	***AZI5***	Lipid transfer protein (*AT4G12510*)
				***MQD22.2***	Lipid transfer protein (*AT5G46890*)
				***EXTA***	Lipid transfer protein (*AT5G46900*)
Lipid binding (GO:0008289, molecular function)	1.61E-05	4.95E-02	51.23	***AZI5***	Lipid transfer protein (*AT4G12510*)
				***MQD22.2***	Lipid transfer protein (*AT5G46890*)
				***EXTA***	Lipid transfer protein (*AT5G46900*)
Down-regulated genes at 12 h post-induction					
Trichoblast differentiation (GO:0010054, biological process)	2.04E-06	1.21E-02	44.77	***COBL9***	Cobra-like protein 9 (*AT5G49270*)
				*LRX1*	Leucine-rich repeat extension-like protein 1 (*AT1G12040*)
				***GT16***	Xyloglucan-specific galacturonosyltransferase 1 (*AT1G63450*)
				***MRH6***	Adenine nucleotide alpha hydrolases -like protein (*AT2G03720*)
Cell development (GO:0048468, biological process)	3.66E-06	7.21E-03	20.83	***COBL9***	Cobra-like protein 9 (*AT5G49270*)
				***LRX1***	Leucine-rich repeat extensin-like protein 1 (*AT1G12040*)
				*NAS1*	Nicotianamine synthase 1 (*AT5G04950*)
				***GT16***	Xyloglucan-specific galacturonosyltransferase 1 (*AT1G63450*)
				***MRH6***	Adenine nucleotide alpha hydrolases-like protein (*AT2G03720*)
Plant-type cell wall (GO:0009505: cellular component)	3.99E-07	4.33E-04	20.17	*LRX1*	Leucine-rich repeat extension-like protein 1 (*AT1G12040*)
				***PER1***	Peroxidase 1 (*AT1G05240*)
				***PER2***	Peroxidase 2 (*AT1G05250*)
				***PER24***	Peroxidase 24 (*AT2G39040*)
				***PME24***	Pectinesterase/pectinesterase inhibitor 24 (*AT3G10710*)
				***PME46***	Pectinesterase/pectinesterase inhibitor 46 (*AT5G04960*)

^*^P-values < 0.05

^**^All FDR values have a *P* < 0.05.* FDR*, false discovery rate

^a^Gene symbols in bold print indicate genes uniquely responsive to C4

At 12-hpi, C4 expression resulted in significant down-regulation of 5 genes associated with cell development with 4 of the DE genes being more specifically associated with trichoblast differentiation (Table 5). Three of the 4 DE genes, *COBRA-LIKE PROTEIN 9* (*AT5G49270, COBL9*), *XYLOGLUCAN-SPECIFIC GALACTURONOSYLTRANSFERASE 1* (*AT1G63450, GT16*) and *ADENINE NUCLEOTIDE ALPHA HYDROLASES-LIKE PROTEIN 6* (*AT2G03720, MRH6*) are regulated by C4, but not by BR*, bes1-D* or *bzr1-1D* (Table 5 and Additional file 3). Moreover, at 12-hpi, 6 enriched DE genes associated cell wall homeostasis were down-regulated, with 5 of the DE genes being responsive to C4, but not to BR*, bes1-D* or *bzr1-1D* (Table 5 and Additional file 3). Taken together, the results suggest that early transcriptional events down-regulated by C4 impact lipid homeostasis, cell development and cell wall integrity independent of the BRSP.

### Differentially expressed miRNAs following C4 induction

MicroRNAs are small non-coding RNAs that are involved in gene regulation of a plethora of biological processes, including development and RNA silencing as an immune response to virus infection [45]. Therefore, a small RNA library analysis was performed to determine early events in regulating miRNA expression following expression of the C4 protein. At 6- and 12- hpi, 11 and 22 DE miRNAs were detected in induced IPC4–28 seedlings, respectively, with 3 being common to both time points (Additional file 5). In control experiments with Sei-0 wild-type seedlings, 1 and 9 DE miRNAs were detected in induced IPC4–28 seedlings, respectively, at 6- and 12-hpi. Five of the 9 DE miRNAs detected at 12-hpi in non-induced IPC4–28 seedlings were also differentially expressed in induced seedling, suggesting a role in developmental regulation.

We used psRNATarget to identify targets of the DE miRNAs. At 12-hpi, miR163 was down-regulated and represents the only DE miRNA that was identified to target transcripts of a C4 regulated DE gene. MiR163 targets *PXMT1*, a member of the Arabidopsis SABATH methyltransferase gene family that prevents seed germination and modulates root development [46]. Both miR163 and PXMT1 are light inducible and there is an inverse correlation between the levels of miR163 and PXMT1 transcripts after C4 induction. The result suggests that up-regulation of *PXMT1* transcript levels, noted above, may, at least in part, be due to the down-regulation of miR163.

### Network analysis

A coorelation network was used to identify key regulator points where DE genes following C4 expression might be involved in modulating metabolic processes (Supplementary Fig. S3, Additional file 1). At 12-hpi, a single protein, TAP2 INTERACTING PROTEIN OF 41 kDa-like (TIP41-like) protein (AT3G54000) was found to act as a hub connecting two large nodes. TIP41 is the orthologue of mammalian and yeast proteins that function in the Target-of-rapamycin (TOR) pathway, which modifies cell growth in response to nutrient status and environmental conditions [47]. In Arabidopsis, TIP41 is constitutively expressed in vascular tissue and has been shown to bind and regulate SERINE/THREONINE PROTEIN PHOSPHATASE 2A (PP2A) activity, a phosphatase that plays a role in numerous signaling pathways regulating growth and development and stress responses [47].

## Discussion

**Fig. 5:**
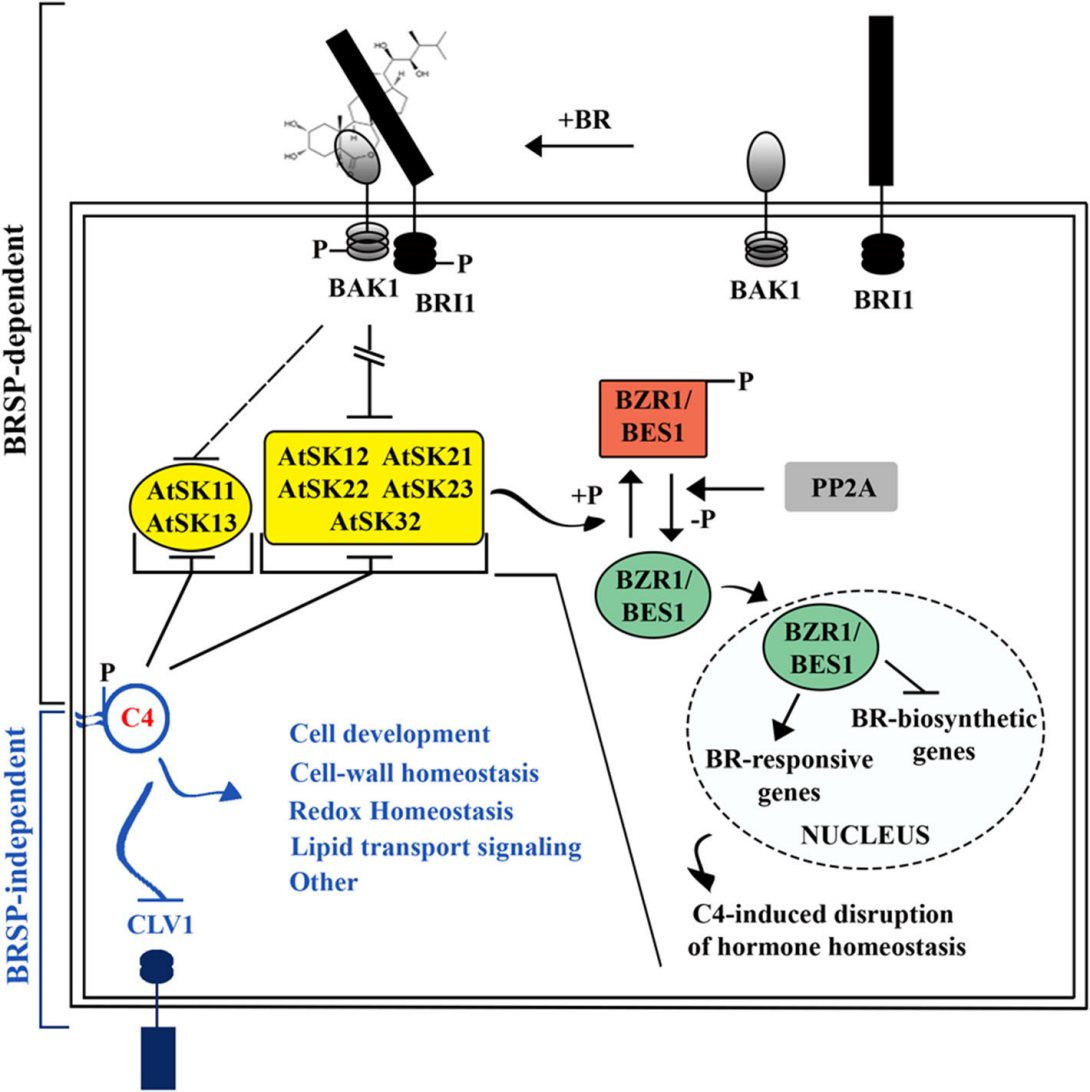
Model showing curtovirus C4 regulated BR-signaling pathway dependent (BRSP-dependent) and BRSP-independent functions at 12-hpi. In the absence of BR, BES1 and BZR1 are phosphorylated by AtSKs, inactivating the transcription factors. In the presence of BR or the BCTV C4 protein, the AtSKs are inactivated, which results in the dephosphorylation and activation of BES1 and BZR1. Activated BES1 and BZR1 locate to the nucleus and control expression of BR-biosynthetic genes and BR-responsive genes. Under constant C4 presence, the result is the disruption of hormone homeostasis. C4 also regulates expression of genes not directly regulated by the BR-signaling pathway (BRSP-independent), having early effects on regulatory pathways affecting cellular processes indicated in blue. The image in this figure was generated by the corresponding author

The BCTV C4 protein is a pathogenic determinate that induces extensive development abnormalities, including oncogenesis. In this study, we looked at early patterns of gene expression following induction of the C4 protein to identify initial events in pathogenesis. Since C4 targets AtSKs [9], we initially assumed that C4-induced gene expression would mimic host-gene expression changes observed in the *bes1-D* and *bzr1-1D* gain-of-function mutants. However, transcriptional changes following induction of the C4 protein only partially mimicked gene expression responses documented for *bes1-D* and *bzr1-1D* (24, 25; Additional file 3). Based on the results we present a working model for early transcriptional changes following C4 expression (Fig. 5). C4 expression results in BRSP-dependent alterations, presumably through the interaction of C4 with AtSKs. However, the results also underscore the importance of C4 altering the *Arabidopsis* transcriptome by one or more BRSP-independent mechanisms.

The expression of DE genes unique to C4 and not regulated by BR*, bes1-*D *and bzr1-1D* could be explained in three ways. First, the C4 protein induces DE genes through interacts with host proteins not involved in the BRSP. The BCTV C4 protein has been shown to interact with CLV1 [29], a protein involved in meristem cell proliferation and differentiation. This BCTV C4-host protein interaction, as well as ones that likely have not been identified, might be expected to have a significant effect on cellular processes and pathogenesis. Second, DE genes unique to C4 expression might arise from C4 differentially regulating AtSK functions not associated with BES1 and BZR1. AtSK11 and AtSK21 regulate enzymes and transcription factors involved in stomatal, root hair and lateral root development, auxin and ABA signaling, tolerance to salt stress and pattern-triggered immunity [14, 18]. Third, since C4 binds to and inhibits AtSKs, it functions downstream of BR, BRI1 and BAK1 signaling at the PM. Therefore, in the absence of BR, BRI1 accumulates [48] and would be available for BR-independent functions; such as, the interaction of BRI1 and BAK1 with G proteins that regulate sugar-responsive growth and development [49]. In addition, BRI1 controls vascular cell fate in *Arabidopsis* root through RECEPTOR-LIKE PROTEIN 44 and phytosulfokine signaling [50]. Therefore, regulatory events upstream of AtSK activities would not be directly impacted by C4 and might, at least in part, explain why some BR-responsive genes are not responsive in the gain-of-function mutants [24, 25].

### Down regulated DE genes are primarily unique to C4

At 12-hpi, 5 enriched GO categories (*lipid transport*, *cell development*, *root epidermal cell differentiation,* and the child term *trichoblast differentiation*, as well as, *plant cell wall*) were identified from the 20 down-regulated DE genes that were uniquely responsive to C4 (Fig. 4, Additional file 3). Four of the down-regulated DE genes are lipid transfer proteins (LTPs), 3 of which have been shown to be up-regulated in the *bzr1-1D* mutant (Additional file 3). The 4 LTPs, are members of the AZI1 family of LTPs [51]. AZI1 and the closely related paralog, EARLY ARABIDOPSIS ALUMINUM INDUCED 1, have been implicated in signaling as positive regulators of systemic acquired resistance (SAR), induced systemic resistance signaling, MAMP-triggered SAR, lignin biosynthesis, root growth under zinc-limiting conditions, and abiotic (salt and cold) tolerance [52–57]. If the DE LTP genes are involved in promoting lipid-based signaling during biotic and abiotic stress, the C4 protein might disrupt this process, such as defense-related signaling, and establish an environment favorable for BCTV infection.

Four down-regulated DE genes were associated with trichoblast differentiation and are involved in root hair development (Table 5). Three of the DE genes, *GOBL9*, *GT16* and *MRH6*, uniquely respond to C4, but not to BR, *bes1-D* or *bzr1-1D* (Additional file 3). COBL9 and GT16, a root hair specific acid xyloglucan that incorporates galacturonic acids residues into root hair cell walls, are required for root hair tip-directed growth [58–60]. MRH6 is required for normal root hair initiation and to restrict development to a single root-hair on each root-hair bearing cell [59]. The fourth DE gene, LEUCINE-RICH REPEAT/EXTENSIN 1 (*LRX1)*, encodes a cell wall localized, chimeric leucine rich-repeat/extension protein that regulates cell wall development and/or polar growth and, subsequently, root hair morphogenesis and elongation [61]. BRs are involved in controlling multiple aspects of root growth and development [62]. Therefore, because expression of C4 mimics high levels of BR by inhibiting AtSK function [9], these findings provide mechanistic relevance to previous phenotypic findings that roots are stunted and root hairs and lateral roots are either absent or developmentally impaired in seedlings ectopically expressing the C4 protein [6]. This suggests that the C4 protein modulates unique alterations to cell development that are independent of those modulated by the BRSP.

At 12-hpi, 6 DE genes associated with the cell wall were down-regulated (Table 5). Five of the DE genes [*PEROXIDASE 1, 2, and 24 (PRX1*, *PRX2*, *PRX24) and PECTIN METHYLTRANSFERASE 24, and 46* (*PME24* and *PME46*)] were uniquely responsive to C4 (Additional file 3). *PRX1*, *PRX2*, and *PRX24* encode class III peroxidases that are primarily involved in cell wall stiffening or loosening [63]. *PRX1* and *PRX2* are paralogs with identical coding sequences. PRX2 is involved in stem lignification in *Arabidopsis*. A PRX2 deficient mutant leads to a significant decrease in total lignin content, altered lignin structure and cell wall thinning in *Arabidopsis* stems [64]. PRX1 is found primarily in roots and leaves and is likely involved in cell wall lignification. It is notable that *GIBBERELLIN 2-OXIDASE 8* (*GA2OX8*) is up-regulation by C4 and BR (Additional file 3) and that up-regulation of *GA2OX8* correlates with decreased lignification in cell walls [65]. While very little is known about the activity of PME24 and PME46, the down-regulation of both PMEs has been associated with cell wall remodeling as has the down-regulation of the DE gene *LRX1* [66]. Although not identified in the GO analysis, *EXTENSIN 13*, a proline-rich cell wall protein, that is uniquely responsive to only C4, was also significantly down-regulated at 12-hpi (Additional file 3). In addition, at 12-hpi, we observed 3 DE genes that are responsive to BR and/or *bes1-D* and *bzr1-1D* with roles in cell wall synthesis that were not identified in the GO analysis, *XYLOGLUCAN ENDOTRANSGLUCOSYLASE/HYDROLASE 19* (*XTH19*), *PME41* and *AT3G10720* (putative member of the PME family). XTH19 has a role in constructing and deconstructing xyloglucans polymers in primary cell walls, while being involved in cell wall construction of growing tissue. XTH19 has also been shown to be involved in cell proliferation of pith tissue during tissue reunion of incised stem [67]. Interestingly, BR has been shown to regulate expression of a plethora of genes involved in cell wall synthesis and remodeling, including genes with a role in protecting cell wall integrity that results from an imbalance of pectin modifications during growth and development [16, 68]. However, at 12-hpi, we did not see regulation of a large number of BR and/or *bes1-D* and *bzr1-1D* responsive DE cell-wall associated genes, an expression pattern that might occur at later times post-C4 expression. The results do suggest that C4 expression regulates the expression of genes involved in cell wall remodeling that are independent of BR-regulated cell wall homeostasis. It is tempting to speculate that the effect of C4 on the cell wall could result in cell wall thinning and remodeling, possible a prerequisite for C4-induced cell division and/or regulation of plasmodesmata molecular exclusion limits for cell-to-cell movement of BCTV. It should be noted that the Tobacco mosaic virus (TMV) movement protein was shown to bind a cell wall associated PME, a requirement for movement of the virus from cell to cell through plasmodesmata [69]. More recently a tobacco PME was shown to enhance RNA silencing induced by TMV, suggesting a role for a PME(s) in a virus defense [70].

### C4-induced alterations to hormone homeostasis primarily depend on regulation of the BRSP

Plant growth and development are highly regulated processes involving the endogenous signaling of multiple plant hormones in complex interconnected networks in response to varying environmental conditions [71]. Since C4 negatively regulates AtSKs, it is not surprising that C4-induced transcriptional changes result in disruptions of hormone homeostasis at 6-hpi, which is enhanced at 12-hpi. Of the 24 up-regulated DE genes affecting hormone homeostasis at 12-hpi (Table 2), only 4 were not responsive to the BRSP, suggesting C4 effects multiple hormone-related processes by primarily regulating the BRSP.

However, two up-regulated DE genes that could affect hormone homeostasis and are responsive to C4, but not BR, *bes1-D* or *bzr1-1D*, are *ACS9* and *LOG5* (Additional file 3). Both directly affect hormone synthesis and levels and could enhance the C4-directed BRSP disruption of hormone homeostasis. *ACS9* was one of three ethylene biosynthesis enzymes (*ACS4*, *5* and *9*) highly enriched as 6- and 12-hpi. Ethylene regulates a number of developmental and stress responses, including seed germination, fruit ripening, senescence, cell elongation and pathogen defense [72]. ACS4, 5 and 9 are closely related type II ACS proteins that are regulated in a ubiquitin-dependent manner and may negatively regulator plant growth in light [73]. BR and cytokinin regulate type II ACS proteins by stabilize the proteins [74]. While the relevance of C4 up-regulating *ACS* genes is not clear, high levels of ethylene might contribute to the stunting of primary and lateral roots observed in transgenic *Arabidopsis* expressing C4 [6]. Many geminiviruses, such as BCTV, are limited to the phloem and replication is restricted to the vascular tissue. Therefore, it is interesting that ethylene promotes cell division during vasculature development in *Arabidopsis* stems [75], a process that ACS9 might take part in or enhance.

LOG5 is one of a 9-member family of cytokinin-activating enzymes in the direct-activation pathway in *Arabidopsis* that converts inactive cytokinin nucleotides to the biologically active free-base forms. Therefore, the up-regulation of *LOG5* at 12-hpi would be expected to have a direct effect on increasing levels of cytokinin. Cytokinin promotes cell division, cell expansion and differentiation, apical dominance and leaf senescence. *LOG5* is expressed in vascular tissue of cotyledons, immature leaves, axillary buds, ovules and roots of *Arabidopsis* [76]. Therefore, cytokinin biosynthesis could be regulated in a vascular-specific, C4-dependent manner during infection, which might be expected to exacerbate the C4-induced BRSP alterations on hormone homeostasis in vascular tissue.

### Defense and redox homeostasis

The primary virus defense responses in plants are thought to be through RNA silencing and resistance (R) genes. In contrast to begomovirus C4/AC4 proteins, there is no evidence that the C4 proteins of curtoviruses are suppressors of RNA silencing and we saw very little evidence that the transcriptional response to C4 was regulated by miRNA activity as early as 12-hpi. However, we cannot exclude the possibility of the DE miRNAs exerting their action through translational repression [77, 78]. Also, begomovirus C4 proteins interact with BAM1 and BAM2 and negatively regulate their role in RNAi spread [30, 31], a function we cannot rule out for BCTV C4. Expression of C4 did up-regulate 14 DE genes related to fungal, bacterial, and oomycete defense (Table 3), but the majority are also responsive to BR and/or *bes1-D* and *bzr1-1D* and may have roles independent of a defense response. Alternatively, extended expression of these pathogen-related genes might benefit BCTV and increase host susceptibility. However, the results do suggest that C4 may disrupting redox homeostasis independent of the BRSP, which might play a role in defense functions [44]. Consistent with C4 modulating redox potential, AtSK11, which BCTV C4 binds [9], phosphorylates and activates glucose-6-phophate dehydrogenase (G6PD). G6PD is required for maintaining cellular redox homeostasis. G6PD activity is reduced in an AtSK11 loss-of-function mutant, resulting in increased levels of reactive-oxygen species and an imbalance in redox potential [79]. Therefore, C4 binding to AtSK11 would be expected to disrupt redox homeostasis.

### Questions on C4-induced hyperplasia

We did not detect C4-induced DE cell cycle-related genes at 6- or 12-hpi in 7-day old seedlings. However, we previously showed that 3 mitotic markers [CYCLIN A1;1 (CYCA1;1), B1;4 (CYCB1;4) and CYCLIN-DEPENDENT KINASE B2:2 (CDKB2;2)], that are up-regulated in the G2/M transition and M phases of the cell cycle [80], were significantly up-regulated by 24-hpi in C4-expressing seedlings relative to non-expressing seedlings [6]. CYCA1;1 and CDKB2;2 are responsive to C4, but not to BR, *bes1-D* or *bzr1-1D* [24, 25]. Similarly, Parks et al. [7] screened transformed *Arabidopsis* plants constitutively expressing Beet severe curly top virus (BSCTV)-C4 for a number of cell-cycle related genes. They found CYCB1;1, CYCB1;3, CYCB2;2, CDKA;1 and CDKB1;1 were up-regulated in BSCTV infected tissue and to a much higher level in BSCTV-C4 expressing transgenic plants. CYCB1;1 and CDKB1;1 are responsive to BSCTV-C4, but not to BR, *bes1-D* or *bzr1-1D* [24, 25]. In addition, CYCLIN-DEPENDENT KINASE INHIBITOR 1 (an inhibitor of the CYCD2;1/CDKA;1 activity), also uniquely responsive to C4, was much more strongly suppressed in C4 expressing plants than in BSCTV infected tissue. Recently, the C4 protein of TLCYnV was shown to interact with *N. benthamiana* NbSKη, sequestering the kinase to the PM [8]. This interaction was suggested to impair NbSKη directed degradation of NbCycD1;1, resulting in accumulation of active NbCycD1;1 and abnormal cell division [8]. However, this may not be a mechanism of action of the BCTV C4 protein in *Arabidopsis* since constitutive overexpression of *CYCD1;1* under regulation of the 35S promoter does not result in a C4 phenotype, but promotes endoreduplication in *Arabidopsis* leaves and hypocotyls [81]. In addition, BR represses *CYCD1;1* expression in *Arabidopsis* [24], although we did not observe C4 mimicking this response as early as 12-hpi.

## Conclusions

A requirement of BCTV C4 pathogenesis is that C4 sequesters AtSKs to the PM [9, 27]. Apparently, regulating the BRSP by sequestering AtSKs to PM is not a unique developmental tool for geminiviruses. OCTOPUS, a PM and vascular localized, cell-specific suppressor of BR-mediated signaling has been shown to promote phloem differentiation by sequestering AtSK21 to the PM [17]. Consistent with this, BES1 and BZR1 are key regulators of phloem and xylem cell differentiation in vascular tissue [82]. The two latter points are significant since BCTV replication is limited to phloem tissue. Our results suggest that BCTV C4-induced pathogenesis alters the balance between growth and development by usurping both the BRSP and one or more BR-independent signaling pathways (Fig. 5). We suggest that C4-pathogenesis results from the protein interacting with and affecting the outcome of a number of metabolic and/or signaling pathways.

## Methods

### Plants and transgene induction

*Arabidopsis thaliana* ecotype Sei-0 (WT) and transgenic Sei-0 line IPC4–28 seedings [6], were used in all experiments. Sei-0 seeds were obtained from Roger Innis (Indiana University, Bloomington, IN) and were originally obtained from the Arabidopsis Information Service seed bank. Line IPC4–28 expresses the BCTV *C4* gene from the regulatory control of a ß-est inducible promoter [6]. For RNA-seq analysis, fifty Sei-0 or IPC4–28 seeds were surface sterilized, place at 4 °C for 2 days, and grown in liquid culture for 7 days at 25 °C with gentle shaking (100 rpm) under continuous light [6]. After 7 days, seedlings were mock-induced or induced with 10 μM ß-est and harvested at 0-, 6- and 12-h post-mock-induction (hpmi) or post-induction (hpi), washed with water, blotted dry, frozen in liquid nitrogen and stored at − 80 °C. Three independent experiments were performed for each *Arabidopsis* line and each treatment.

### RNA extraction, RNA-seq and qRT-PCR

RNA-seq results were obtained from RNA extracted from 3 independent biological replicates. Seedlings grown in liquid culture with or without induction with ß-est were homogenized in 0.75 ml of Trizol Reagent in ZR BashingBead Lysis tubes (2.0 mm beads; Zymo Research, Irvin, CA, U.S.A.) on a Geno/Grinder (SPEX SamplePrep 2010, Metuchen, NJ, U.S.A.) for 60 s. at 1750 rpm. RNA was extracted using a Direct-zol RNA Miniprep kit, which included a DNase I on-column treatment, as described by the manufacturer (Zymo Research, Irvin, CA, U.S.A.). Initial quality control of the RNA samples was performed using an Agilent 2100 Bioanalyzer (Agilent, Santa Clara, CA, U.S.A.) to determine RNA purity, concentration and integrity. Sample concentration was normalized in 25uL of nuclease-free H_2_O prior to library preparation. Libraries were prepared and sequenced by the Georgia Genomics and Bioinformatics Core Lab team (GGBC). Briefly, libraries were prepared using KAPA’s Stranded mRNA-seq kit (cat. KK4821). During library preparation, mRNA was selected using oligo-dT beads, the RNA was fragmented, and cDNA was generated using random hexamer priming. Single indices and Illumina adapters were ligated to make the sequencing libraries. Quality control of the libraries were performed using a Qubit and Fragment Analyzer to determine library concentration and size distribution. Libraries were multiplexed using an equimolar amount and the concentration of the final pool was assessed using qPCR. The final pool was sequenced on an Illumina NextSeq 500 platform using the paired-end (PE) 75 protocol.

The small RNA (sRNA) libraries were prepared and sequenced by the GGBC. Briefly, sRNA libraries were prepared using the Bioo Scientific NEXTFLEX Small RNA-Seq Kit v3 with the following steps: 3′ and 5′ adapters were selectively ligated to the small RNA molecules, and cDNA was synthesized using a primer specific to the 3′ adapter sequence. Index sequences were added to each library during library PCR. Final libraries were size selected to enrich for small RNA products using the bead ratios described in the kit’s protocol with NEXTFLEX Clean-up Beads. Quality control of the prepared libraries was performed using the Qubit and Fragment Analyzer to determine the concentration and size of the libraries. SRNA libraries were multiplexed using equimolar ratios. The final pool was sequenced on the Illumina NextSeq 500 platform using single-end (SE) 75 protocol.

RNA-seq raw reads were quality filtered and trimmed using Trim Galore (Babraham Bioinformatics – Trim Galore) before mapping to the *Arabidopsis* reference genome (TAIR10) using the short reads quasi-mapper Salmon [83]. Genes that had a total of 10 reads or less across all samples were filtered out. PCA analysis from the package DESeq2 was used to assess the quality of the samples and replicates. The count matrix was normalized and fitted using the default DESeq2 normalization method. Differential analysis was carried out on the normalized matrix using the contrast experimental design (Fig. 1a) using DESeq2 [84].

SRNA-seq raw reads were quality filtered and trimmed using the filter tool in the UEA small RNA Workbench [85]. This step includes removing rRNA and mRNA degradation products as well as low complexity reads and adapter traces. Alignment of small RNA reads to the *Arabidopsis* sRNA transcriptome, reads count, normalization and differential expression analyses were done using the tools available in the UEA small RNA workbench. The miRNA-mRNA target analysis was done using the psRNATarget tool [86] using TAIR10 reference. Genes or miRNA that were significantly up-regulated or down-regulated following ß-est induction of the C4 protein in transgenic IPC4–28 seedlings relative to mock-induced control seedlings were designated as differentially expressed (DE) if they had log2 fold changes ≥2.0 with adjusted *p*-values ≤0.05.

For RT-qPCR analysis, RNA extracted and purified for RNA-seq was used, representing three independent biological replicates. cDNAs were synthesized using SuperScript™ III Reverse Transcriptase (Invitrogen) and primers describe in Additional file 6. PCRs were performed on a QuantStudio 5 Real-Time PCR System (Applied Biosystems) using SYBR™ Green PCR Master Mix (Applied Biosystems). *MONENSIN SENSITIVITY 1* (*MON1, AT2G28390*) was used as an endogenous housekeeping control. A relative standard curve method was used to calculate the fold change of gene expression between ß-est induced and mock induced transgenic IPC4–28 plants. C4-induced transcriptional changes were normalized to gene expression in noninduced seedlings.

### Network analysis

The network analysis was conducted in three steps: 1) filtration of low count genes and logarithmic transformation, 2) differential expression analysis, and 3) co-expression network construction. Only genes with read counts in all three time points (0-, 6-, and 12-hpi) were used in this analysis. We used the Bioiconductor ‘org. At.tai.db’ to annotate genes [87]. Differential expression analysis was conducted using DESeq2 [84]. Differentially expressed genes with *P*-value > 0.05 were removed. Gene-Gene similarity matrix was developed using Pearson correlation and Euclidean distances measures. The similarity matrix was converted to adjacency matrix using power transformation [88]. The adjacency matrix was visualized in the Cytoscape software. Only edges with a minimum of 0.75 coefficient score were permitted in the network. Both edges and nodes were colored to reflect gene-gene correlation coefficiency and connectivity.

## Supplementary Information

**Additional file 1: Supplementary Figure S1.** A histogram comparing the counts of quality-trimmed reads and mapped reads for each biological replicate (Rep1, Rep2, and Rep3) from transgenic (Trans) or wild-type (WT) seedlings at 0-, 6-, and 12-hours post-induction (hpi) or hours post-mock induction (hpmi) as well as the overall mapping rate. I, induced. NI, not induced. **Supplementary Figure S2.** A principal component analysis (PCA) of the replicates using the read count in the gene-condition count matrix. The PCA shows clustering of replicates and the variance/distance between 6- and 12-h post-induction (hpi) of C4Trans_I replicates and replicates of all other conditions. Trans, transgenic seedlings. WT, wild-type seedlings. I, induced. NI, not induced. Hpmi, hours post-mock induction. **Supplementary Figure S3.** Co-expression network of DE genes. Differentially expressed genes at 12 h post-induction. Nodes are colored based the node degree of connectivity and edges are colored based on correlation coefficient values.

**Additional file 2: Table S1.** List of significant differentially expressed genes at 6- and 12-hpi and list of average C4 mRNA reads/sample.

**Additional file 3: Table S2.** Responsiveness of C4 differentially expressed genes at 12-hpi relative to brassinosteroid and *bes1-D* and *bzr1-1D* gain-of-function mutants.

**Additional file 4: Table S3.** Gene ontology (GO) terms indicating over-represented, up-regulated genes in hormone homeostasis at 6 h post-induction.

**Additional file 5: Table S4.** Significantly expressed miRNAs.

**Additional file 6: Table S5.** Primers used for RT-qPCR.

## Data Availability

The datasets generated and analyzed during the current study are available in the Genbank repository, Accession: PRJNA701411. Access at https://www.ncbi.nlm.nih.gov/Traces/study/?acc=PRJNA701411&o=acc_s%3Aa

## Abbreviations

ABCG40: ABC TRANSPORTER G FAMILY 40
ACS: 1-AMINOCYCLOPROPANE-1-CARBOXYLATE SYNTHASE
AtSK: *Arabidopsis thaliana* shaggy-like protein kinase
AZI: AZELAIC ACID INDUCED
BAK1: BRASSIONSTEROID INSENITIVE 1-ASSOCIATED RECEPTOR KINASE 1
BAM: BARELY ANY MERISTEM
BCTV: Beet curly top virus
BES1: BRI1-EMS SUPPRESSOR 1
BR: Brassinosteroid
BRI1: BRASSINOSTEROID INSENSITIVE 1
BRSP: Brassinosteroid-signaling pathway
BSCTV: Beet severe curly top virus
BZR1: BRASSINAZOLE-RESISTANT 1
CDK: Cyclin dependent kinase
CLV1: CLAVATA 1
CML41: Calmodulin-like 41
COBL9: COBRA-LIKE PROTEIN 9
CYC: Cyclin
DE: Differentially expressed
G6PD: Glucose-6-phosphate dehydrogenase
GA2OX8: GIBBERELLIN 2-OXIDASE 8
GGBC: Georgia Genomics and Bioinformatics Core Laboratory
GO: Gene ontology
GT16: GALACTURONOSYLTRANSFERASE 1
HIR1: HYPERSENSITIVE INDUCED REACTION 1
IPC4–28: Inducible promoter C4–28
LRX1: LEUCINE-RICH REPEAT/EXTENSIN 1
LOG5: LONELY GUY 5
LTP: Lipid transfer protein
MRH6: ADENINE NUCLEOTIDE ALPHA HYDROLASE-LIKE PROTEIN 6
MON1: MONENSIN SENSITIVITY 1
NbCYCD1:1: *Nicotiana benthamiana* cyclin D1.1
NbSKη: *Nicotiana benthamiana* shaggy-like protein kinase eta
PCA: Principle component analysis
PM: Plasma membrane
PME: PECTIN METHYLTRANSFERASE
PP2A: SERINE/THREONINE PROTEIN PHOSPHATASE 2A
PRX: Peroxidase
RT-qPCR: Reverse transcription quantitative polymerase chain reaction
PXMT1: PARAXANTHINE METHYLTRANSFERASE 1
SAR: Systemic acquired resistance
TIP41: TAP2 interacting protein of 41
TMV: Tobacco mosaic virus
TLCYnV: Tomato leaf curl Yunnan virus
TUB2: TUBULIN β CHAIN
XTH19: XYLOGLUCAN ENDOTRANSGLUCOSYLASE/HYDROLASE 19
WT: Wild type
