# Supplementary material for: Early transcriptome changes induced by the Geminivirus C4 oncoprotein: setting the stage for oncogenesis
Source: BMC Genomics. 2021 Mar 2;22:147. doi: 10.1186/s12864-021-07455-y (PMC7923490; doi:10.1186/s12864-021-07455-y)
Supplement: Supplementary file 1 — Additional file 1: Supplementary Figure S1. A histogram comparing the counts of quality-trimmed reads and mapped reads for each biological replicate (Rep1, Rep2, and Rep3) from transgenic (Trans) or wild-type (WT) seedlings at 0-, 6-, and 12-hours post-induction (hpi) or hours post-mock induction (hpmi) as well as the overall mapping rate. I, induced. NI, not induced. Supplementary Figure S2. A principal component analysis (PCA) of the replicates using the read count in the gene-condition count matrix. The PCA shows clustering of replicates and the variance/distance between 6- and 12-h post-induction (hpi) of C4Trans_I replicates and replicates of all other conditions. Trans, transgenic seedlings. WT, wild-type seedlings. I, induced. NI, not induced. Hpmi, hours post-mock induction. Supplementary Figure S3. Co-expression network of DE genes. Differentially expressed genes at 12 h post-induction. Nodes are colored based the node degree of connectivity and edges are colored based on correlation coefficient values. [file 12864_2021_7455_MOESM1_ESM.docx]

**Supplementary Figures**

**Early transcriptome changes induced by the Geminivirus C4 oncoprotein: setting the stage for oncogenesis**.

**C. Michael Deom**^1*^, **Magdy S. Alabady**^2^ **and Li Yang**^1^

^1^Department of Plant Pathology, University of Georgia, Athens, GA, United States.

^2^Department of Plant Biology, University of Georgia, Athens, GA, United States.

^*^Correspondence: [deom@uga.edu](mailto:deom@uga.edu)

Supplementary Figure S1. A histogram comparing the counts of quality-trimmed reads and mapped reads for each biological replicate (Rep1, Rep2, and Rep3) from transgenic (Trans) or wild-type (WT) seedlings at 0-, 6-, and 12-hours post-induction (hpi) or hours post-mock induction (hpmi) as well as the overall mapping rate. I, induced. NI, not induced.

Supplementary Figure S2. A principal component analysis (PCA) of the replicates using the read count in the gene-condition count matrix. The PCA shows clustering of replicates and the variance/distance between 6- and 12-hours post-induction (hpi) of C4Trans_I replicates and replicates of all other conditions. Trans, transgenic seedlings. WT, wild-type seedlings. I, induced. NI, not induced. Hpmi, hours post-mock induction.

Supplementary Figure S3. Co-expression network of DE genes. Differentially expressed genes at 12 hours post-induction. Nodes are colored based the node degree of connectivity and edges are colored based on correlation coefficient values.
