# Supplementary material for: Early transcriptome changes induced by the Geminivirus C4 oncoprotein: setting the stage for oncogenesis
Source: BMC Genomics. 2021 Mar 2;22:147. doi: 10.1186/s12864-021-07455-y (PMC7923490; doi:10.1186/s12864-021-07455-y)
Supplement: Supplementary file 3 — Additional file 3: Table S2. Responsiveness of C4 differentially expressed genes at 12-hpi relative to brassinosteroid and bes1-D and bzr1-1D gain-of-function mutants. [file 12864_2021_7455_MOESM3_ESM.docx]

| Table S2. Responsiveness of C4 differentially expressed genes at 12-hpi relative to brassinosteroid and *bes1-D* and *bzr1-1D* gain-of-function mutants. | | | | |
| --- | --- | --- | --- | --- |
| **Locus** | **Log2 fold**  **Change** | **Gene Symbol** | **BR***^**^*  **-responsive** | ***bes1-D*/*bzr1-1D****^**^* **-responsive** |
|  |  | **BR signaling pathway-dependent** |  |  |
| *AT1G12040* | -4.24 | *LRX1* | Down | - |
| *AT5G04950* | -3.31 | *NAS1* | Down | - |
| *AT4G25780* | -3.01 | *CAP* | Down | - |
| *AT5G06870* | 2.74 | *PGIP2* | Up | Up/Down |
| *AT1G28190^*^* | 2.81 | *Hypothetical protein* | - | Up/- |
| *AT2G44080^*^* | 2.88 | *ARL* | Up | - |
| *AT3G54030* | 2.94 | *BSK6* | Up | Up/- |
| *AT3G54000* | 3.02 | *TIP41-like protein* | Up | Up/Up |
| *AT5G01210^*^* | 3.04 | *HXXXD-type acyl-transferase protein* | Up | Up/- |
| *AT4G16515* | 3.14 | *RGF6* | Up | Up/Up |
| *AT1G76680^*^* | 3.15 | *OPR1* | Up | - |
| *AT3G10720* | 3.12 | *Pectin methylesterase inhibitor* | Up | Up/- |
| *AT5G09970* | 3.20 | *CYP78A7* | Up | Up/- |
| *AT5G64770^*^* | 3.22 | *RGF9* | Up | Up/- |
| *AT3G10720* | 3.26 | *Pectin methylesterase inhibitor* | Up | Up/- |
| *AT3G03840* | 3.27 | *SAUR27* | Up | -/Up |
| *AT4G38850^*^* | 3.49 | *SAUR15* | Up | Up/Up |
| *AT1G69490* | 3.62 | *NAC029* | Up | - |
| *AT5G50335^*^* | 3.64 | *Hypothetical protein* | Up | Up/- |
| *AT4G23030^*^* | 3.65 | *DTX49* | Up | Up/- |
| *AT1G24140* | 3.70 | *3MMP* | Up | - |
| *AT4G02330^*^* | 3.70 | *PME41* | Up | Up/- |
| *AT2G17040^*^* | 3.83 | *NAC036* | Up | - |
| *AT1G15580* | 3.89 | *IAA5* | Up | Up/Up |
| *AT2G34930* | 3.93 | *Disease resistance protein/LRR* | Up | Up |
| *AT1G26380* | 3.99 | *FOX1* | Up | Up/- |
| *AT4G14365^*^* | 3.99 | *XBAT34* | Up | Up/- |
| *AT3G50770* | 4.04 | *CML41* | Up | Down/- |
| *AT3G22060^*^* | 4.12 | *CRRSP38* | Up | - |
| *AT4G30290^*^* | 4.14 | *XTH19* | Up | Up/Up |
| *AT2G44130^*^* | 4.24 | *KFB39* | Up | - |
| *AT4G15360* | 4.28 | *CYP705A3* | - | Up/- |
| *AT4G39830^*^* | 4.29 | *Cupredoxin superfamily protein* | Up | Up/- |
| *AT4G36110^*^* | 4.29 | *SAUR9* | Up | /-Up |
| *AT3G14380* | 4.30 | *CASPL2A2* | Up | - |
| *AT4G21200^*^* | 4.53 | *GA2OX8* | Up | - |
| *AT5G65800^*^* | 4.62 | *ACS5* | Up | -/Up |
| *AT4G23220^*^* | 4.63 | *CRK14* | Up | - |
| *AT2G32680^*^* | 4.65 | *RLP23* | Up | - |
| *AT2G18010^*^* | 4.71 | *SAUR10* | Up | Up/Up |
| *AT2G22810^*^* | 4.71 | *ACS4* | Up | - |
| *AT4G23150* | 4.72 | *CRK7* | Up | - |
| *AT3G57240* | 4.79 | *BG3* | Up | - |
| *AT5G25190^*^* | 4.98 | *ERF003* | Up | Up/Down |
| *AT5G24110^*^* | 5.28 | *WRKY30* | Up | - |
| *AT1G68320^*^* | 5.64 | *MYB62* | - | Up/- |
| **BR signaling pathway-independent** | | | | |
| *AT4G12510^*^* | -6.36 | AZI5 | Up | -/Up |
| *AT5G46890^*^* | -4.88 | *Lipid transfer protein* | - | -/Up |
| *AT5G46900^*^* | -4.65 | *Lipid transfer protein* | - | -/Up |
| *AT5G35190* | -4.06 | *EXT13* | - | - |
| *AT1G27140* | -4.06 | *GSTU14* | - | - |
| *AT1G05240* | -4.01 | *PRX1* | - | - |
| *AT1G05250* | -4.01 | *PRX2* | - | - |
| *AT2G03720* | -3.96 | *MRH6* | - | - |
| *AT2G39040^*^* | -3.93 | *PRX24* | - | - |
| *AT5G62330* | -3.92 | *Hypothetical protein* | - | - |
| *AT3G60280* | -3.91 | *UCC3* | - | - |
| *AT5G24880^*^* | -3.88 | *Chromo domain cec-like protein* | - | - |
| *AT3G10710* | -3.83 | *PME24* | - | - |
| *AT5G04960* | -3.82 | *PME46* | - | - |
| *AT5G49270* | -3.65 | *COBL9* | - | - |
| *AT3G21340* | -3.59 | *Leucine-rich repeat protein kinase* | - | - |
| *AT2G47540* | -3.53 | *Pollen Ole e 1 allergen* | - | - |
| *AT4G12520* | -3.48 | *Lipid transfer protein* | Up | -/Up |
| *AT1G63450* | -3.43 | *GT16* | - | - |
| *AT1G70880* | -3.38 | *Polyketide cyclase/dehydrase* | - | - |
| *AT1G75300* | 2.96 | *isoflavone reductase-like* | - | - |
| *AT4G24275^*^* | 3.03 | *Hypothetical protein* | - | - |
| *AT5G18470^*^* | 3.20 | *Curculin-like lectin family protein* | - | - |
| *AT4G35190* | 3.24 | *LOG5* | - | - |
| *AT5G14360* | 3.48 | *Ubiquitin-like superfamily protein* | - | - |
| *AT1G21520* | 3.55 | *Hypothetical protein* | - | - |
| *AT1G66700* | 3.69 | *PXMT1* | - | - |
| *AT4G28703* | 3.73 | *RmlC-like cupins superfamily protein* | Down | -/Down |
| *AT5G52760* | 3,74 | *Copper transport protein family* | - | - |
| *AT5G44575* | 3.90 | *Hypothetical protein* | - | - |
| *AT5G38250* | 3.93 | *Protein kinase family protein* | - | - |
| *AT2G30770* | 4.04 | *CYP71A13* | - | - |
| *AT1G15520^*^* | 4.19 | *ABCG40* | - | Down/- |
| *AT1G69920* | 4.19 | *GSTU12* | - | - |
| *AT5G25180* | 4.30 | *CYP71B14* | - | - |
| *AT5G49690* | 4.42 | *UDP-Glycosyltransferase protein* | - | - |
| *AT2G29470^*^* | 4.45 | *GSTU3* | - | - |
| *AT5G40900* | 4.48 | *Nucleotide-diphospho-sugar transferase* | - | - |
| *AT2G43580^*^* | 4.52 | *Chitinase family protein* | - | - |
| *AT1G26945^*^* | 4.55 | *PRE6* | - | - |
| *AT2G34655* | 4.66 | *Hypothetical protein* | - | - |
| *AT3G49700^*^* | 4.71 | *ACS9* | - | - |
| *AT5G57520^*^* | 4.83 | *ZFP2* | - | - |
| *AT3G44560* | 5.02 | *FAR8* | - | - |
| *AT3G04300^*^* | 5.20 | *RmlC-like cupins superfamily protein* | - | - |
| *AT1G23730^*^* | 5.23 | *BCA3* | - | - |
| *AT2G44070^*^* | 5.23 | *NagB/RpiA/CoA transferase-like* | - | - |
| *AT2G33130^*^* | 5.80 | *RALF18* | - | - |

All log2 fold changes had adjusted p-values ≤0.05.

^*^ Indicate DE genes common to both 6- and 12-hpi.

*^**^*Obtained from 24, 25.
