# Supplementary material for: Early transcriptome changes induced by the Geminivirus C4 oncoprotein: setting the stage for oncogenesis
Source: BMC Genomics. 2021 Mar 2;22:147. doi: 10.1186/s12864-021-07455-y (PMC7923490; doi:10.1186/s12864-021-07455-y)
Supplement: Supplementary file 4 — Additional file 4: Table S3. Gene ontology (GO) terms indicating over-represented, up-regulated genes in hormone homeostasis at 6 h post-induction. [file 12864_2021_7455_MOESM4_ESM.docx]

| Table S3. Gene ontology (GO) terms indicating over-represented, up-regulated genes in hormone homeostasis at 6 hours post-induction. | | | | | |
| --- | --- | --- | --- | --- | --- |
| GO terms | P-value | FDR | Fold enrichment | Gene^*^  symbol | Gene name (Gene ID) |
| Ethylene biosynthetic  (GO:0009693) | 1.25E-05 | 5.67E-03 | 74.01 | *ACS4*  *ACS5*  ***ACS9*** | *1-aminocyclopropane-1-carboxylate synthase 4 (AT2G22810)*  *1-aminocyclopropane-1-carboxylate synthase 5 (AT5G65800)*  *1-aminocyclopropane-1-carboxylate synthase 9 (AT3G49700)* |
| Regulation of hormone levels  GO:0010817) | 3.16E-06 | 2.34E-03 | 11.09 | *ACS4*  *ACS5*  ***ACS9***  *GA2OX8*  *MYB62*  *RGF9*  *WAG1* | *1-aminocyclopropane-1-carboxylate synthase 4 (AT2G22810)*  *1-aminocyclopropane-1-carboxylate synthase 5 (AT5G65800)*  *1-aminocyclopropane-1-carboxylate synthase 9 (AT3G49700)*  *Gibberellin 2-oxidase (AT4G21200)*  *R2R3-MYB transcription family 62 (AT1G68320)*  *Root meristem growth factor 9 (AT5G64770)*  *Serine/threonine protein kinase WAG1 (AT1G53700)* |
| Response to auxin  GO:0009733) | 2.32E-06 | 1.96E-03 | 11.63 | *ACS4*  *ARGOS*  *SAUR9*  *SAUR10*  *SAUR15*  *XTH19*  *WAG1* | *1-aminocyclopropane-1-carboxylate synthase 4 (AT2G22810)*  *Auxin-regulated gene involved in organ growth (AT3G59900)*  *Small auxin upregulated RNA 9 (AT4G36110)*  *Small auxin upregulated RNA 10 (AT2G18010)*  *Small auxin upregulated RNA 15 (AT4G38850)*  *Xyloglucan endotransglucosylase/hydrolase protein 19 (AT4G30290)*  *Serine/threonine protein kinase WAG1 (AT1G53700)* |
| Response to hormone  GO:0009725) | 9.64E-10 | 1.42E-06 | 6.44 | *ACS4*  *ASC5*  ***ABCG40***  *ARL*  *ARGOS*  *CRRSP38*  *ERF003*  *MYB62*  *OPR1*  *PME41*  *SAUR9*  *SAUR10*  *SAUR15*  *WAG1*  *WRKY30*  *XTH19* | *1-aminocyclopropane-1-carboxylate synthase 4 (AT2G22810)*  *1-aminocyclopropane-1-carboxylate synthase 5 (AT5G65800)*  *ABC transporter G family member 40 (AT1G15520)*  *ARGOS-like protein (AT2G44080)*  *Auxin-regulated gene involved in organ growth (AT3G59900)*  *Cysteine-rich repeat secretory protein 38 (AT3G22060)*  *Ethylene-responsive transcription factor 003 (AT5G25190)*  *R2R3-MYB transcription family 62 (AT1G68320)*  *12-oxophytodienoate reductase 1 (AT1G76680)*  *Pectin methylesterase 41 (AT4G02330)*  *Small auxin upregulated RNA 9 (AT4G36110)*  *Small auxin upregulated RNA 10 (AT2G18010)*  *Small auxin upregulated RNA 15 (AT4G38850)*  *Serine/threonine protein kinase WAG1*  *(AT1G53700)*  *WRKY transcription factor 30 (AT5G24110)*  *Xyloglucan endotransglucosylase/hydrolase protein 19 (AT4G30290)* |

P-values <0.05. FDR, false discovery rate, with P<0.05.

^*^ Gene symbols in bold print indicate genes uniquely responsive to C4.
