# Supplementary material for: Early transcriptome changes induced by the Geminivirus C4 oncoprotein: setting the stage for oncogenesis
Source: BMC Genomics. 2021 Mar 2;22:147. doi: 10.1186/s12864-021-07455-y (PMC7923490; doi:10.1186/s12864-021-07455-y)
Supplement: Supplementary file 6 — Additional file 6: Table S5. Primers used for RT-qPCR. [file 12864_2021_7455_MOESM6_ESM.docx]

| Table S5. Primers used for RT-qPCR | |
| --- | --- |
| Gene | Primers |
| *AT5G04950* | TCCCCACCAAGATTGCCTTC (FP)  CGAGTGTGTTTGCGTGTGAG (RP) |
| *AT1G05250* | TGCCCGCCTACAGATTTCAG (FP)  AGCCCTTTCTTCTGAGCCAC (RP) |
| *AT5G46890* | AGAGCTACCACAAGAAGCCC (FP)  CAACAGTTGGACGTTGGTGG (RF) |
| *AT2G47540* | TGGTTACGAAGCGGAGGATG (FP)  GAAACGCCCTGCACTCTTTG (RP) |
| *AT5G25190* | ACGACCACAACAACGCTTTC (FP)  ATCCTCCGCTGTCTCAAACG (RP) |
| *AT2G44130* | CGTTCCAATTCCAATCCGCC (FP)  GAGAAGCTCTGTTTTGCCGC (RP) |
| *AT3G50770* | atctcacgaggcagctcaag (FP)  ATCGCCATCAACTTCACCGT (RP) |
| *AT3G57240* | TACACTCTGTTCACCGCACC (FP)  ACGATCTCCAACGAACCACC (RP) |
| *AT5G65800* | TGGCGATGCTTTCCTTTTGC (FP)  TGTTGCAGAGCTGATTCCGT (RP) |
| *AT2G30770* | TCCCTCAGTCTCAGGTACGG (FP)  ACCTCTTGAGCTGCTTCACC (RP) |
| *TUB2* | AGCAATACCAAGATGCAACTGCG (FP)  TAACTAAATTATTCTCAGTACTCTTCC (RP) |
| *AT2G28390* | AACTCTATGCAGCATTTGATCCACT (FP)  TGATTGCATATCTTTATCGCCATC (RP) |

FP, forward primer. RP, reverse primer.
